# Systems Pharmacology Based Strategy for Q-Markers Discovery of HuangQin Decoction to Attenuate Intestinal Damage

**DOI:** 10.3389/fphar.2018.00236

**Published:** 2018-03-20

**Authors:** Xiao-min Dai, Dong-ni Cui, Jing Wang, Wei Zhang, Zun-jian Zhang, Feng-guo Xu

**Affiliations:** ^1^Key Laboratory of Drug Quality Control and Pharmacovigilance, Ministry of Education, China Pharmaceutical University, Nanjing, China; ^2^State Key Laboratory of Natural Medicines, China Pharmaceutical University, Nanjing, China; ^3^College of Pharmacy, Shaanxi University of Chinese Medicine, Xianyang, China; ^4^State Key Laboratory for Quality Research in Chinese Medicines, Macau University of Science and Technology, Taipa, Macau

**Keywords:** Q-marker, systems pharmacology, traditional Chinese medicine, HuangQin decoction, intestinal damage

## Abstract

The quality control research of traditional Chinese medicine (TCM) is lagged far behind the space of progress in modernization and globalization. Thus the concept of quality marker (Q-marker) was proposed recently to guide the quality investigations of TCM. However, how to discover and validate the Q-marker is still a challenge. In this paper, a system pharmacology based strategy was proposed to discover Q-marker of HuangQin decoction (HQD) to attenuate Intestinal Damage. Using this strategy, nine measurable compounds including paeoniflorin, baicalin, scutellarein, liquiritigenin, norwogonin, baicalein, glycyrrhizic acid, wogonin, and oroxylin A were screened out as potential markers. Standard references of these nine compounds were pooled together as components combination according to their corresponding concentration in HQD. The bioactive equivalence between components combination and HQD was validated using wound healing test and inflammatory factor determination experiment. The comprehensive results indicated that components combination is almost bioactive equivalent to HQD and could serve as the Q-markers. In conclusion, our study put forward a promising strategy for Q-markers discovery.

## Introduction

Traditional Chinese medicine (TCM) plays a vital role in prevention and treatment of diseases and receives more and more attention (Jiang et al., 2010). Due to its highly complex chemical composition, TCM is confronting a major challenge in quality control research (Yang et al., 2017). In Chinese Pharmacopeia monographs, the quality standards of TCM were usually established based on the absolute quantitation of one or several specific chemical compounds. This approach can only ensure the consistency of the assigned chemical markers. It is often questionable whether these chemical markers are responsible for and directly related to the holistic efficacy of TCM. Many efforts have been made to drive the advance of quality control research of TCM (Tilton et al., 2010; Long et al., 2015; Wang F. et al., 2017). But the proposed strategy or methods are quite complex and not easy to follow. Thus, a standardized and commonly accepted strategy for TCM quality control research is needed. Recently, the concept of quality marker (Q-marker) was proposed to standardize TCM quality research and to enhance the quality consistency (Liu et al., 2016). However, how to discover and validate the Q-marker is still a huge challenge.

**FIGURE 1:**
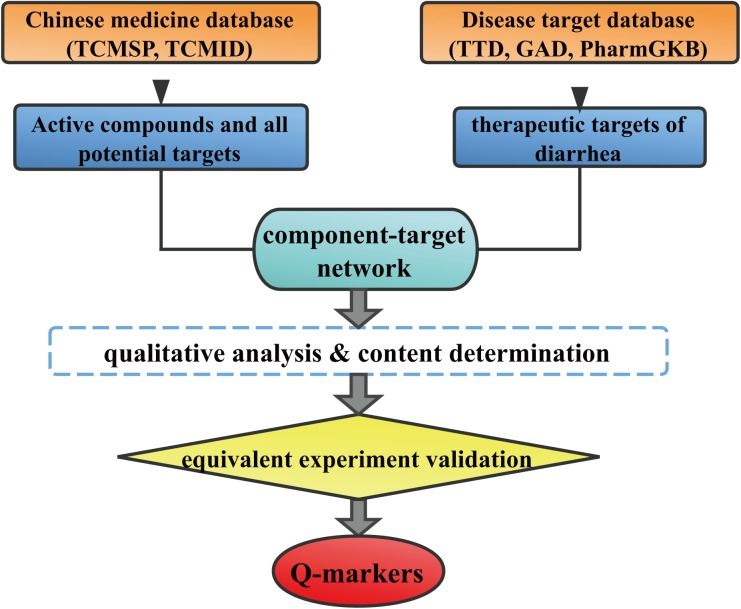
Whole framework of Q-markers discovery of TCM based on systems pharmacology and a case of HQD.

Systems pharmacology is an emerging approach that integrates chemoinformatics, network pharmacology and -omics data. It is a useful tool to achieve a comprehensive insight into the therapeutical mechanism of multi-compound herbs. The public availability of system pharmacology platforms and other bioinformation databases put systems pharmacology-based TCM research strategy into practice (Ru et al., 2014). It has been successfully used to reveal the material basis and the mechanism of Yin-Huang-Qing-Fei capsule (Yu et al., 2017) and rhubarb (Xiang et al., 2015) on the treatment of chronic bronchitis and renal interstitial fibrosis, respectively. Systems pharmacology provides a bridge to link the TCM chemical constituents with the corresponding targets, which would facilitate the Q-markers discovery. Therefore, we proposed a systems pharmacology based strategy (**Figure 1**) for Q-marker discovery. The feasibility of this strategy was tested by taking HuangQin decoction (HQD) as an example.

HuangQin decoction (HQD) is a basic formula listed in *Treatise on Exogenous Febrile Disease* written by Zhongjing Zhang. It has been widely used in China for more than 1800 years on the treatment of gastrointestinal (GI) ailments, including diarrhea, abdominal spasms, vomiting, and nausea (Wang X. et al., 2017). Recent studies have revealed that PHY906, a modified formulation derived from HQD, could ameliorate chemotherapy-induced GI toxicity and enhance the therapeutic efficacy of irinotecan, capecitabine and other antitumor drugs (Lam et al., 2010; Saif et al., 2010; Wang et al., 2011). HQD is constituted with four medicinal herbs, i.e., *Scutellaria baicalensis* Georgi, *Glycyrrhiza uralensis* Fisch, *Paeonia lactiflora* Pall, and *Ziziphus jujuba* Mill. Up to now, it is still unclear that which ingredients of HQD are active to ameliorate intestinal damage, which significantly limits the establishment of quality standards.

In this paper, we tried to discover the Q-marker of HQD to treat diarrhea using a system pharmacology based strategy. After collecting the active components of HQD and the corresponding therapeutic targets of diarrhea, the component-target (C-T) network was constructed firstly. LC-IT-TOF/MS fingerprint was used to clarify which active compounds actually existed in water decoction of HQD. Then the detectability of selected potential markers were tested and the absolute concentration of measurable components were quantified using HPLC/UV. Bioactive equivalent experiment was performed to evaluate the efficacy of HQD and the combination of selected potential markers from the aspect of alleviating intestinal damage.

## Materials and Methods

### Chemicals and Reagents

Paeoniflorin, baicalin, scutellarein, liquiritigenin, baicalein, glycyrrhizic acid, wogonin, and oroxylin A were purchased from Chengdu Herbpurify Co., Ltd. (Sichuan, China). Glycyrrhizic acid ammonium salt and norwogonin were purchased from ChemFaces (China). All other reagents and solvents were of high performance liquid chromatography (HPLC) grade. Deionized water was purified using a Milli-Q system (Millipore, Bedford, MA, United States). *Scutellaria baicalensis* Georgi (Hebei Province), *Glycyrrhiza uralensis* Fisch (Inner Mongolia of China), *Paeonia lactiflora* Pall (Anhui Province), and *Ziziphus jujuba* Mill (Henan Province) were authenticated by Dr. Ehu Liu (State Key Laboratory of Natural Medicines, China Pharmaceutical University, China).

### Collection of Active Ingredients and Diarrhea Targets

The ingredients of the four constitutional herbs, i.e., *Scutellaria baicalensis* Georgi, *Glycyrrhiza uralensis* Fisch, *Paeonia lactiflora* Pall, and *Ziziphus jujuba* Mill in HQD were collected from Traditional Chinese Medicines for Systems Pharmacology Database and Analysis Platform (TCMSP^1^), Traditional Chinese Medicine integrative database (TCMID), TCM Database @ Taiwan, HIT and wide-scale literature mining. Generally, ADME screening was used in previous prediction, which includes series of pharmacokinetic parameters such as oral bioavailability (OB), drug-likeness (DL), and blood–brain barrier (BBB) value (Shen et al., 2016). Considering that our study focused on intestinal damage, the active components might exert therapeutic effect without being absorbed into serum or brain. Thus, we only set DL ≥ 0.05 as criteria to filter out active ingredients as many as possible.

Targets of all effective components in HQD were collected from DrugBank, TCMSP, and STITCH. The targets that are in close relationship with diarrhea were obtained from PharmGKB^2^, TTD database^3^ (Yang et al., 2016) GAD (Genetic Association Database) and OMIM (Online Mendelian Inheritance in Man, up data to 2017). Then UniProt database^4^ was employed to standardize the target related genes and to focus on the targets from the human. The genes that are associated with diarrhea and can be targeted by HQD were kept. And then, the Compound-Target (C-T) network were generated and their topological properties were analyzed by Cytoscape 3.4.0.

### Active Components Identification in HQD by LC/MS

LC-IT-TOF/MS was used to clarify which active compounds are actually existing in water decoction of HQD. One milliliter of standard decoction HQD (Wang X. et al., 2017) was dissolved in a suitable amount of 80% (v/v) methanol with the assistant dissolve effect of DMSO by ultra-sonication and subsequently centrifuged (16000 rpm, 4°C) for 10 min. The supernatants were filtered and analyzed on a ZORBAX SB-C18 rapid resolution HT (2.1 mm × 100 mm, 1.8 μm) (Agilent Technologies). The mobile phase consisted of 0.1% formic acid (A) and methanol (B). The gradient elution began with 10% B, increased to 45% B in 12 min, further increased to 100% B in 16 min and last for 6 min, and brought back to 10% B in 1 min followed by 10 min of re-equilibration. The mass spectrometry (MS) analysis was performed in a ultrafast LC-ion trap time-of-flight mass spectrometer via electrospray ionization (ESI) interface (SHIMADZU, Japan). The parameters were as follows: ESI (±), nebulizing gas rat, 1.5 L/min; drying gas pressure, 100 kPa; detector voltage, 1.85 kV; interface voltage, -3.5 kV; CDL and heat block temperature, 200°C; ion accumulation time, 30 ms. The mass range was set at m/z 100–1000. The components in HQD were identified by comparing with the reference standards available in our lab or the fragment models in literatures. The results were combined with those identified in PHY906 (Ye et al., 2007).

### Potential Markers Quantification in HQD by HPLC/UV

After checking the identified components of HQD in the C-T network, only the common ones were screened out as potential markers. The detectability of these selected potential markers was tested and the absolute concentration of measurable components were quantified using HPLC/UV. After dilution and filtering, HQD was analyzed on an Agilent 1100 series HPLC system (Agilent, United States) using Agilent Zorbax SB-C18 column (250 mm × 4.6 mm, 5 μm). The mobile phase consisted of 0.1% phosphoric acid in water (A) and acetonitrile (B). The gradient elution program was 19–21% B at 0–8 min, 21–35% B at 8–10 min, 35–35% B at 10–18 min, 35–40% B at 18–20 min, 40–40% B at 20–38 min, 40–100% B at 38–43 min. The flow rate was kept at 1.0 ml/min at 30°C. Different detection wavelengths were set for different compounds. 236 nm for paeoniflorin; 278 nm for baicalin, baicalein, wogonin, liquiritigenin; 250 nm for glycyrrhizic acid ammonium salt; 270 nm for oroxylin A; 340 nm for scutellarein; 280 nm for norwogonin. Standard references of these compounds were pooled together as components combination according to their corresponding concentration in HQD.

### Bioactive Equivalence Assessment Between Components Combination and HQD

#### Cell Culture

Lipopolysaccharide (LPS)-stimulated NCM460 damage (Bhattacharyya et al., 2008) and LPS-stimulated THP-1-derived macrophage inflammation (Perezperez et al., 1995) were used as two cell models to assess the bioactive equivalence between components combination and HQD. NCM460 and THP-1 were obtained from Model Animal Research Center of Nanjing University and Stem Cell Bank of Chinese Academy of Sciences, respectively. Cells were grown at 37°C under a humidified atmosphere with 5% (v/v) CO_2_. NCM460 and THP-1 were cultured in Dulbecco’s modified Eagle’s medium (DMEM) (Boster Biological Technology Co., Ltd.) and Roswell Park Memorial Institute (RIPM) 1640 medium (Gibco-Thermo Fisher Scientific, United States) respectively, containing 10% fetal bovine serum and 1% penicillin-streptomycin (Biological Industries, Israel).

#### Cell Migration Assay

Collective migration of epithelial cells refers to fundamental physiological processes as an inherent part of embryonic morphogenesis, cancer and wound healing, which can be measured by scratch assay (Das et al., 2015). In colon epithelial monolayer (NCM460), opening of a free surface by scratch-wounding triggers collective movement of the surrounding cells to fill the gap. To assess the effect of medicines on NCM460, we pretreated cells with HQD, components combination and baicalin (corresponding concentrations in 400, 200, 100, 50 μg/ml HQD) for 24 h. After pre-incubation and the 100% cell confluent observed, scratch-wounding was performed. The supernatant was removed and the cells were washed with PBS three times to remove the damaged cells. Then cells were subjected to 1 μg/ml LPS (Lipopolysaccharides from *Escherichia coli* O111:B4, Sigma) for 24 h except the vehicle control group. The images of two migrating epithelial monolayers of NCM460 was captured with an inverted phase contrast microscope (Nikon Eclipse Ti-U), which was used to calculate the % relative cell migration according to the following equation (Buranasukhon et al., 2017).

$$\%\text{Relative migration=}\frac{{\text{Area between cells}}_{\text{0 h}}-{\text{Area between cells}}_{24\text{ h}}}{{\text{Area between cells}}_{\text{0 h}}}\times 100$$

#### TNF-α and PGE_2_ Release

To assess the anti-inflammatory effect of HQD, components combination and baicalin, the NCM460 and THP-1-derived macrophages were pretreated for 12 h with medicines. Then, NCM460 were cultured in serum-free DMEM supplemented with LPS(1 μg/ml) for 6 or 12 h, while THP-1-derived macrophages were cultured in serum-free RIPM 1640 supplemented with 1 μg/ml LPS (8 h for TNF-α, 21 h for PGE_2_) (Padilla et al., 2017). The accumulated TNF-α and PGE_2_ in the culture medium were measured using commercial ELISA kits [Multisciences(Lianke) Biotech for TNF-α and MEIMIAN for PGE_2_] according to the manufacturer’s instruction.

### Statistical Analysis

All data were expressed as mean ± standard deviation (SD). Data were subjected to statistical analysis using Graphpad Prism 5.0 (Graphpad Software, San Diego, CA, United States). One-way analysis of variance (ANOVA) with Dunnett’s *post hoc* test was carried out for statistical comparison. In all cases, the value of *P* < 0.05 was considered to be statistical significance.

## Results

### Collection of HQD Ingredients and Diarrhea Targets

Considering that intestinal tissues and intestinal content play important roles in the occurrence of diarrhea, we selected DL as the only standard to filter active ingredients. The name and Mol ID of 186 ingredients from *Scutellaria baicalensis* Georgi, 111 from *Paeonia lactiflora* Pall, 236 from *Glycyrrhiza uralensis* Fisch, and 226 from *Ziziphus jujube* Mill was shown in **Supplementary Table S1**. The corresponding target that these ingredients act on was screened out based on TCMSP and STITCH database (**Supplementary Table S2**). At the same time, 64 diarrhea-related proteins were found from PharmGKB, TTD, GAD, and OMIM (**Supplementary Table S3**). Only 33 common targets from these two independent search were kept, which were interacted with 208 ingredients of HQD (**Supplementary Table S4**).

### Compound-Target Network Construction and Analysis

**FIGURE 2:**
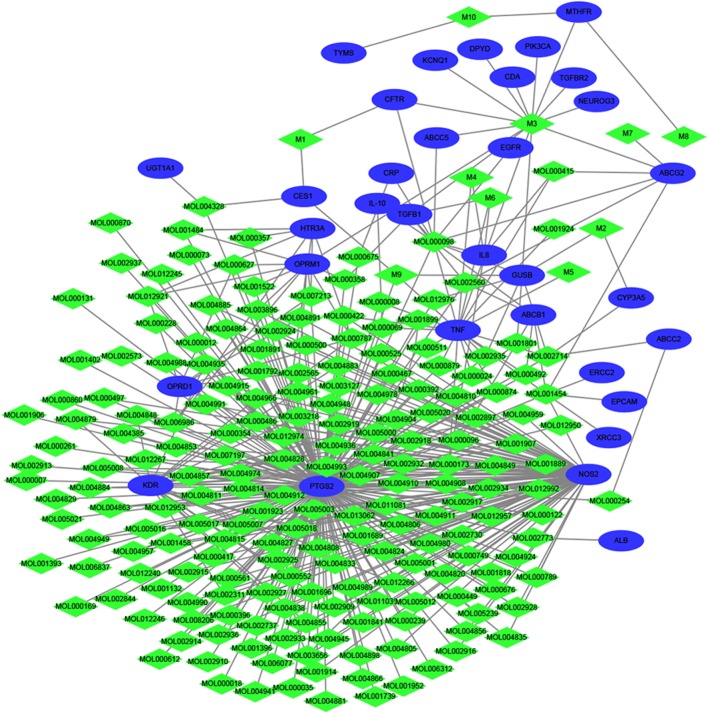
Therapeutic compound-target network. The green diamond nodes represent compounds, and blue ellipses are targets.

As small molecules typically exert their bioactive effects through interactions with protein targets. Thus in order to identify the interaction between the filtered 208 compounds and 33 diarrhea targets, a network was established. As we can see from **Figure 2**, 430 compound-target interactions were generated. The node degree represents the connectedness of a node with other nodes and it is the basic quantitative properties of network. The degree of compounds and targets were listed in **Supplementary Table S5**. Among these 33 targets, Prostaglandin G/H synthase 2 (PTGS2, *D* = 199) has the highest degree, followed by Nitric (nitric) oxide synthase (NOS2, *D* = 97), Vascular endothelial growth factor receptor 2 (KDR, *D* = 25), Tumor necrosis factor (TNF, *D* = 20) and so on, which indicated that they played a significant role in the network as the hub target. Wogonin, oroxylin A, and berberine could interacted with PTGS2 and NOS2 simultaneously. Rutin, wogonin, baicalein, and paeoniflorin could interacted with TNF. The above results clearly elucidated the “multi-component and multi-target” mechanism of HQD and synergistic therapeutical effect on diarrhea.

### Active Components Identification and Quantification of HQD

Although above results suggested that 208 ingredients have effects on diarrhea-related targets, it does not mean that all these 208 components are detectable in HQD. The phytochemical components in water decoction of the four constitutional herbs were then identified by LC-IT-TOF/MS fingerprint in ESI positive and negative ion modes (**Supplementary Figure S1**). Totally, 38 compounds in HQD were identified by comparison with available reference standards in our lab or the fragment information in literatures, including 8 from *Glycyrrhiza uralensis* Fisch, 2 from *Paeonia lactiflora* Pall and 28 from *Scutellaria baicalensis* Georgi (**Supplementary Table S6**). Combining these 38 compounds with those identified in PHY906 (Ye et al., 2007), we got 79 compounds. Eleven of them that could be well matched with the C-T network of diarrhea were kept as potential markers.

Quantitative determination results (**Supplementary Figure S2**) demonstrated that except chrysin and rutin, the content of the rest 9 potential markers in HQD was more than Limit of Quantitation (LOQ). The LOQ of chrysin and rutin by HPLC/UV was 66.15 and 45.14 ng, respectively. Therefore, paeoniflorin, baicalin, scutellarein, liquiritigenin, norwogonin, baicalein, glycyrrhizic acid, wogonin, and oroxylin A were screened out as potential markers. Standard references of these 9 compounds were pooled together as components combination according to their corresponding concentration in HQD.

### Bioactive Equivalence Assessment Between Components Combination and HQD

Wound healing test and inflammatory factor determination experiment results indicated that HQD showed remarkable protective effects and components combination exerted the same or better effects.

**FIGURE 3:**
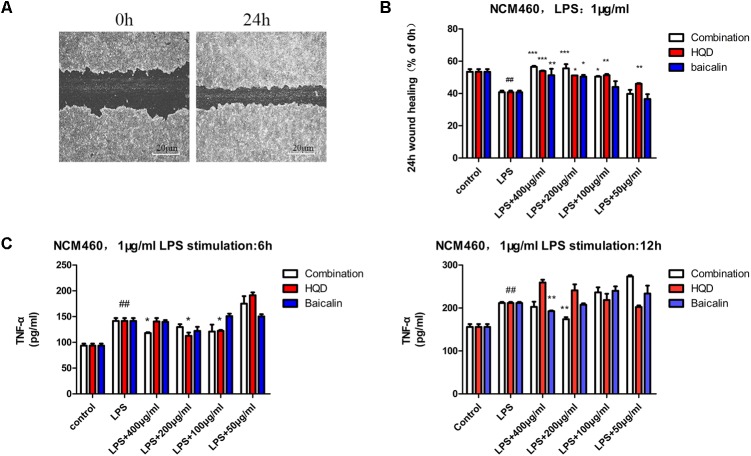
Activity assays of HQD, components combination and baicalin in NCM460. **(A)** Phase contrast microscopy images of two migrating epithelial monolayers of NCM460. **(B)** NCM460 were incubated with vehicle, HQD, the combination and baicalin for 24 h, following cell scratch and 24 h stimulation of LPS. 24 h wound healing was determined as % of 0 h. **(C)** NCM460 were pretreated with vehicle, HQD, the combination and baicalin for 12 h, following 6 or 12 h stimulation of LPS, the accumulated TNF-α in the culture medium were measured using commercial ELISA kits. Results are expressed as mean ± SD of at least three independent experiments. ^##^*P* < 0.01 versus control group, ^∗^*P* < 005, ^∗∗^*P* < 0.01, ^∗∗∗^*P* < 0.001 versus model group (One-way analysis of variance with Dunnett’s *post hoc* test).

Representative phase-contrast images of control group wound areas at 0 and 24 h following scratching were shown in **Figure 3A**. Quantitative results demonstrated that LPS stimulation resulted in significantly lower cell mobility of NCM460 than the control group (*P* < 0.01). Components combination increased the cell mobility of LPS-stimulated NCM460 with dose-dependent and the effect was better than that of HQD at the same dose (**Figure 3B**). Baicalin (12 μg/mL), one of the most abundant compounds in HQD, showed some activities but could not achieve bioactive equivalence with HQD at the same dose level (400 μg/mL). In addition, LPS stimulation resulted in a substantial increase of TNF-α secretion in NCM460, while pre-incubation of components combination or HQD alleviated the LPS-induced increase of TNF-α. The results of LPS stimulation 6 h suggested that 400 μg/mL components combination had a similar efficacy to 200 μg/mL HQD. With LPS stimulation 12 h, only 200 μg/mL components combination exerted notable anti-inflammatory effect, which demonstrated that the anti-inflammatory effect of components combination is superior to that of HQD (**Figure 3C**). Baicalin exerted weak effects and the results were consistent with theory of superimposed effect in TCM.

**FIGURE 4:**
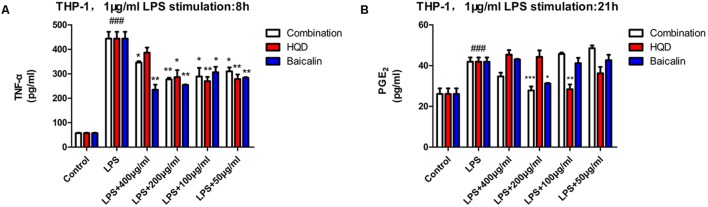
Effects of HQD, components combination and baicalin on TNF-α and PGE_2_ release in THP-1. **(A)** THP-1 was pretreated with vehicle, HQD, the combination and baicalin for 12 h, following 8 h stimulation of LPS. The accumulated TNF-α in the culture medium were measured using commercial ELISA kit. **(B)** THP-1 was incubated with vehicle, HQD, the combination and baicalin for 12 h, following 21 h stimulation of LPS. Supernatant of THP-1 cells was collected for PGE_2_ production assay by commercial kit. Results are expressed as mean ± SD of at least three independent experiments. ^###^*P* < 0.01 versus control group, ^∗^*P* < 0.05, ^∗∗^*P* < 0.01, ^∗∗∗^*P* < 0.001 versus model group (One-way analysis of variance with Dunnett’s *post hoc* test).

To further investigate the anti-inflammatory action on macrophages, effects of components combination and HQD on TNF-α and PGE_2_ production in LPS-activated THP-1 were determined. Differentiated THP-1 was obtained by 48 h treatment with phorbol 12-myristate 13-acetate (PMA). Stimulation of LPS for 8 h increased TNF-α release, whereas preincubation with HQD or components combination notably alleviated the elevation of TNF-α compared with model group. At the optimum concentration 200 μg/mL, components combination showed comparable effect with 100 or 50 μg/mL HQD. An interesting finding is that baicalin showed the best activity compared with components combination and HQD, which could be used to explain the monarch role of *Scutellaria baicalensis* Georgi in HQD (**Figure 4A**). Stimulation of LPS for 21 h significantly increased PGE_2_ production, pre-treatment with 200 μg/mL components combination or 100 μg/mL HQD had the same effect to alleviate the LPS-induced increase of PGE_2_. Baicalin showed some activity but it was inferior to HQD (**Figure 4B**).

## Discussion

Traditional Chinese medicine show advantage especially on the treatment of chronic disease, and receive more and more attention. However, the quality control problem of TCM is a major obstacle hindering its modernization and globalization. Thus the concept of Q-marker was proposed recently to guide the TCM quality investigations. The Q-marker of TCM refers to a group of bioactive constituents that are closely associated with the therapeutic effects. The bottleneck in Q-marker-based quality standard investigation is how to screen out the chemical markers. Zhang et al. (2016a) tried to find the potential Q-markers of *Corydalis* Rhizoma based on biosynthesis, specificity, and pharmacodynamics experiments. Compounds that could be found in the brain tissues were regarded to exert antalgic effect. Non-targeted metabolomics and artificial nerve network were employed to explore the identity markers for five different parts of *P. ginseng* (Qiu et al., 2016). But the bioactivity of these markers was not taken into account. A triarchic theory of “property-effect-component” and multidiscipline-based strategies are proposed to discover effect-associated markers. The key steps are to test the effect of the extract or single compounds on multiple models and to determine the pharmacokinetics parameters (Zhang et al., 2016b). It is obvious that the process is time-consuming due to the complex composition of herbs. Although, significant progress has been made for Q-marker discovery, there are some drawbacks in the current studies. There still needs to be a standardized and commonly accepted strategy to follow.

Therefore, in this paper we proposed a systems pharmacology-based Q-marker discovery strategy. This strategy, integrating target prediction databases of Chinese medicine and disease databases, facilitates our understanding of effective components and was successfully applied to the study of HQD. As a result, 9 compounds were filtered out as potential markers, which interacted with 10 diarrhea-related targets including PTGS2, NOS2, and TNF etc. Previous studies have revealed that PHY906, the modified formulation derived from HQD, performed its effect on the intestinal toxicity by inhibiting PTGS2, NOS2, and TNF (Lam et al., 2010). These results proved the feasibility of our strategy to some extent.

Another huge challenge in Q-marker investigation is how to validate whether the selected Q-marker could be responsible for the holistic efficacy of TCM. Thus we borrow the concept of bioactive equivalent combinatorial compounds. Standard references of the selected compounds were pooled together as components combination according to their corresponding concentration in HQD. Irinotecan caused NCM460 damage was chosen as a model to study the bioactive equivalence between components combination and HQD. At the first stage, we only use the cell survival rate as parameter. The result was disappointed and HQD showed no effect on cell survival rate (**Supplementary Figure S3**), which was incompatible with *in vivo* experiment (Wang X. et al., 2017). It was speculated that cell survival rate was not a sensitive parameter and a mechanism based experiment should be designed. According to the C-T network of HQD on the treatment of diarrhea, we found that wogonin, norwogonin, and oroxylin A could affect PTGS2 and NOS2 activity simultaneously, liquiritigenin, baicalin, baicalein, and scutellarein were also associated with PTGS2. Previous studies have revealed that paeoniflorin, baicalin, baicalein, and wogonin could decrease production of tumor necrosis factor-α (TNF-α) (Kwak et al., 2014; Zhai and Guo, 2016). LPS could stimulate intestinal damage and increase the expression of inflammatory factor at the same time (Chen et al., 2001; Huang et al., 2007) in spite of little influence on NCM460 cell survival rate (**Supplementary Figure S4**). Thus, LPS-stimulated NCM460 damage (Bhattacharyya et al., 2008) and LPS-stimulated THP-1-derived macrophage inflammation (Perezperez et al., 1995) were used as cell models to perform the bioactive equivalence assessment using cell mobility, TNF-α and PGE_2_ release as sensitive parameters.

## Conclusion

The discovery and validation of Q-marker still face enormous challenges despite the fact that the concept of Q-marker has been presented and great efforts have been made. In this study, a systems pharmacology based strategy was proposed to discover Q-markers of TCM. Compared with other approaches to establish Q-markers, systems pharmacology contributes to finding the effect-associated markers faster and takes full advantage of the existing data. Using this strategy, nine compounds in HQD were screened out to compose components combination. The components combination has been validated to be almost bioactive equivalent to original decoction and could be deemed as the Q-markers of HQD. It is promising that systems pharmacology could be applied to Q-marker discovery to ensure efficacy and batch-to-batch consistency of TCM. The limitation of this study was that the contribution of each component has not been clarified, which emphasized the value of further research.

## Author Contributions

X-mD carried out most of the studies, performed the statistical analysis, and wrote the manuscript. D-nC performed the composition identification experiment of HQD. JW and WZ provided professional advice. Z-jZ and F-gX designed the study and revised the manuscript. All authors gave approval to the final version.

## Conflict of Interest Statement

The authors declare that the research was conducted in the absence of any commercial or financial relationships that could be construed as a potential conflict of interest.

## Footnotes

1 http://lsp.nwu.edu.cn/tcmsp.php

2 https://www.pharmgkb.org/

3 http://bidd.nus.edu.sg/group/cjttd/

4 http://www.uniprot.org/

## Supplementary Material

FIGURE S1 Fingerprint chromatography of HQD.

FIGURE S2 Quantitative determination results of potential Q-markers.

FIGURE S3 Influence of CPT-11 and HQD on NCM460 cell survival rate.

FIGURE S4 Influence of LPS and HQD on NCM460 cell survival rate.

TABLE S1 Constituents of herbs in HQD.

TABLE S2 All targets of constituents in HQD.

TABLE S3 All 64 targets related to diarrhea.

TABLE S4 Active constituents of herbs in HQD and their corresponding targets related to diarrhea.

TABLE S5 The degree of compounds and targets.

TABLE S6 The identified components of HQD in our lab.
