# Supplementary figures and images for: Systems Pharmacology Based Strategy for Q-Markers Discovery of HuangQin Decoction to Attenuate Intestinal Damage
Source: Front Pharmacol. 2018 Mar 20;9:236. doi: 10.3389/fphar.2018.00236 (PMC5870050; doi:10.3389/fphar.2018.00236)

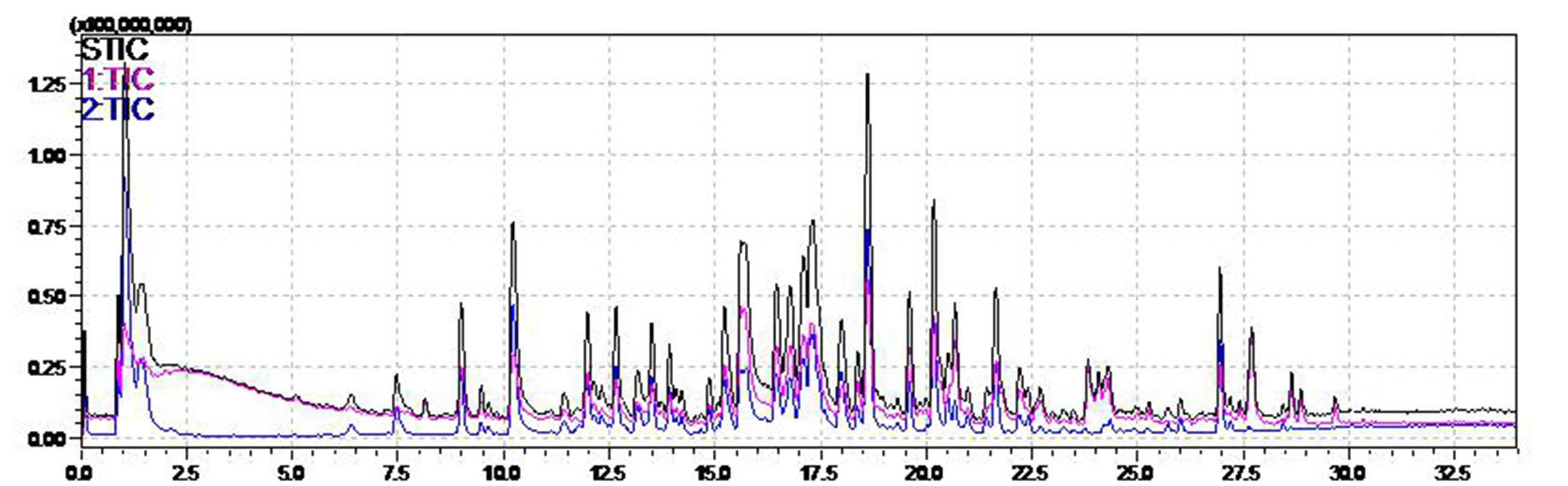

Supplement: FIGURE S1 — Fingerprint chromatography of HQD. [file Image_1.TIF]

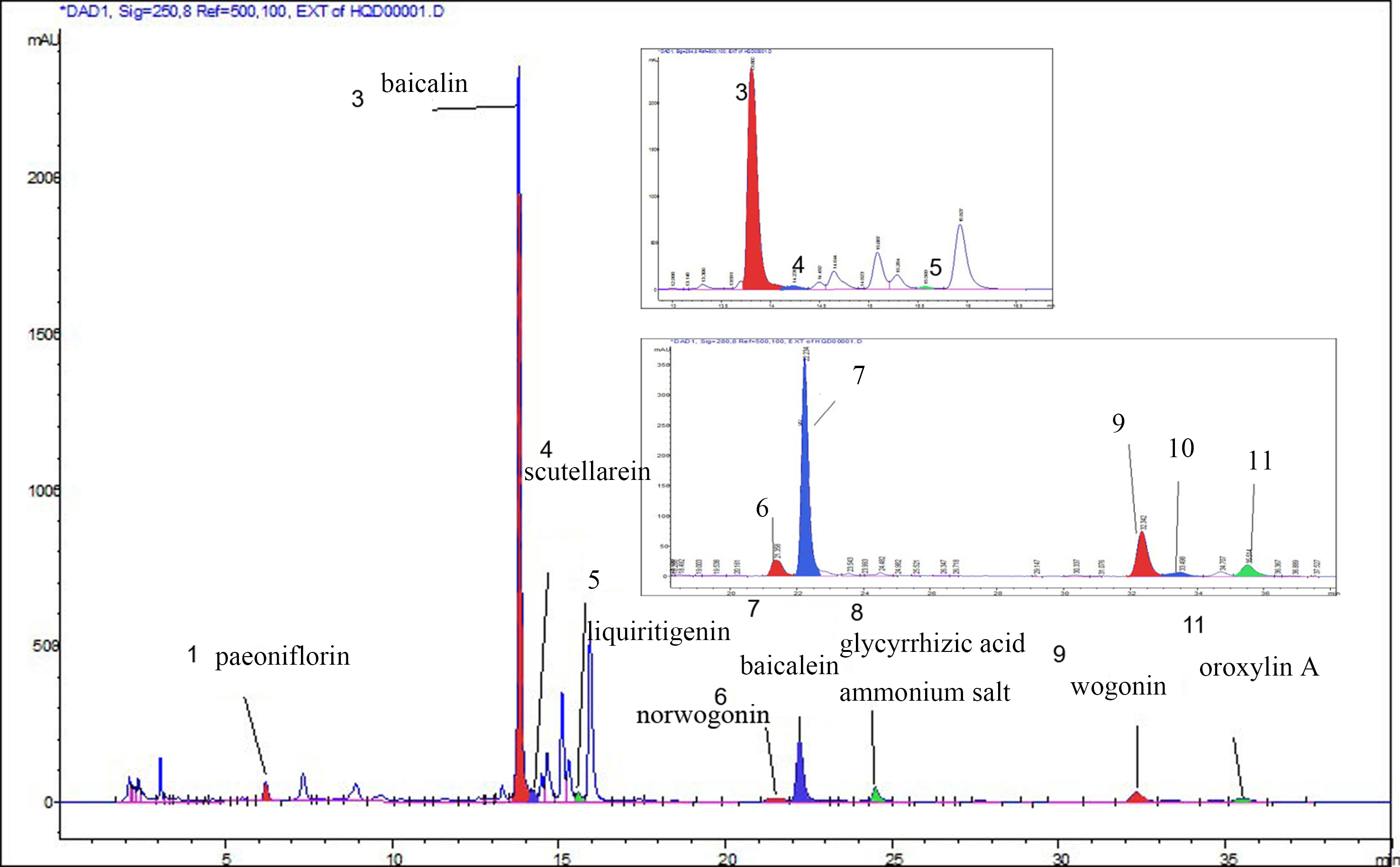

Supplement: FIGURE S2 — Quantitative determination results of potential Q-markers. [file Image_2.TIF]
